# Letter from Dr. Smith

**Published:** 1848-01

**Authors:** 


TERRE HAUTE, DEC. 18, 1847.

DOCT. TAYLOR,

DEAR SIR: I received the first No. of the Dental Register in due time, and at the end of my communication, I find certain inquiries proposed by yourself, relating to the cases which I reported.

First, whether the teeth have since become carious. To this I will answer, that in almost every instance there has been a tendency to decay rapidly.

The fluids of the mouth appear to be much more acrid, and unhealthy than usual, and tartar deposited in greater quantities.

At the time of first treatment, the children generally had little or no tartar around the teeth, but since, there has been a disposition to more or less accumulations of it.

The chronic diarrhœa referred to, has invariably attended those cases where the teeth remained loose, and in most cases the teeth get more firm as the diarrhœa is overcome by treatment. Still several of the patients, on whom the disease has become very extensive and malignant, have more or less loose teeth, and the teeth will probably never become very firmly fixed in the alveola, for the periosteum around the teeth appears to be entirely destroyed by inflammation.

I have so far been able to overcome the disease, by a strict general and local treatment. Still I think that the teeth and gums will never fully recover from the effects of the disease.

In several instances, the teeth present a dark and lifeless appearance, resembling those ulcerated at the root. At present, I have no case of this type under treatment. The cold weather has had a beneficial tendency, in arresting it, showing conclusively to my mind, that the general system was deeply involved in the disease.

Respectfully,

H. R. SMITH.

